# Engineered Bacteriorhodopsin May Induce Lung Cancer Cell Cycle Arrest and Suppress Their Proliferation and Migration

**DOI:** 10.3390/molecules26237344

**Published:** 2021-12-03

**Authors:** Chui-Wei Wong, Ling-Ning Ko, Hung-Jin Huang, Chii-Shen Yang, Shan-hui Hsu

**Affiliations:** 1 Institute of Polymer Science and Engineering, National Taiwan University, Taipei 10617, Taiwan; caryn1111@gmail.com (C.-W.W.); helix70258@gmail.com (H.-J.H.); 2 Department of Biochemical Science and Technology, National Taiwan University, Taipei 10617, Taiwan; lilikoi7508@gmail.com (L.-N.K.); csy.ntu@gmail.com (C.-S.Y.); 3 Institute of Cellular and System Medicine, National Health Research Institutes, Miaoli 35053, Taiwan; 4 Research and Development Center for Medical Devices, National Taiwan University, Taipei 10617, Taiwan

**Keywords:** optogenetic protein, non-small cell lung cancer, cytotoxicity, proton pump, migration ability

## Abstract

Highly expressible bacteriorhodopsin (HEBR) is a light-triggered protein (optogenetic protein) that has seven transmembrane regions with retinal bound as their chromophore to sense light. HEBR has controllable photochemical properties and regulates activity on proton pumping. In this study, we generated HEBR protein and incubated with lung cancer cell lines (A549 and H1299) to evaluate if there was a growth-inhibitory effect with or without light illumination. The data revealed that the HEBR protein suppressed cell proliferation and induced the G_0_/G_1_ cell cycle arrest without light illumination. Moreover, the migration abilities of A549 and H1299 cells were reduced by ~17% and ~31% after incubation with HEBR (40 μg/mL) for 4 h. The Snail-1 gene expression level of the A549 cells was significantly downregulated by ~50% after the treatment of HEBR. In addition, HEBR significantly inhibited the gene expression of Sox-2 and Oct-4 in H1299 cells. These results suggested that the HEBR protein may inhibit cell proliferation and cell cycle progression of lung cancer cells, reduce their migration activity, and suppress some stemness-related genes. These findings also suggested the potential of HEBR protein to regulate the growth and migration of tumor cells, which may offer the possibility for an anticancer drug.

## 1. Introduction

The *Halophilic archaea (haloarchaea)* are grown in aerobic heterotrophs that dominate hypersaline environments. Hypersaline conditions, such as salt lakes, salt ponds, and solar saltern facilities, are usually exposed to strong sunlight, which leads to the higher salinity than that of sea water [1,2]. To protect the damage from light energy and high salinity, the *haloarchaea* generate a proton gradient system through the photo-reactive rhodopsin proteins [3]. The first discovered microbial rhodopsins (M-Rho) are typically seven-pass transmembrane helix region bound with retinal to absorb light energy for ion translocation or phototaxis response [4,5]. The photochemical properties of ion-translocating rhodopsins fall into several categories, such as the light-driven outward proton transport bacteriorhodopsin (BR) [4,6], the light-driven inward chloride transport halorhodopsin (HR) [7,8], outward sodium transport (KR2) [9], and the nonselective cation channel channelrhodopsin II (ChR2) [10,11].

Bacterial rhodopsin (BR), a highly stable light-driven proton pump, can excrete proton out of the cell through light perception and regulate the ion balance of the cell [12,13]. In the early stage, BR protein was mostly produced from the slow-growing extreme halophilic microorganisms and few from genetically recombinant *Escherichia coli* (*E. coli*). However, the low availability and high cost of BR protein limited its potential of bioengineering applications and commercialization. In a previous study, the high-yield, functional, and thermally stable BR recombinant protein was successfully produced [14]. This photosensitive membrane protein, named highly expressible bacteriorhodopsin (HEBR), was generated in *E. coli* using a mutated bacteriorhodopsin (BR) from *Haloarcula marismortui* (HmBRI/D94N) [15], and its structure and biochemical effects were thoroughly evaluated [14,15]. This particular HEBR responded to light with an absorption wavelength at 532 nm (green light) by photocycle measurement and its capability of pumping proton anions outward under light exposure [16]. According to our earlier study, the engineered light-sensitive BR proteins can serve as a trigger to influence the mammalian neural stem cells (NSCs) under light exposure [16]. Besides, another type of optogenetic protein, HR, can hyperpolarize or inhibit action potentials during the period of after hyperpolarization in neuron cells [17]. The light-activated proteins are frequently used to investigate the regulation of neural activity in mammalian cells. However, there are only a few studies on the effect of light-sensitive BR proteins on cancer cell behavior under light exposure.

Epithelial to mesenchymal transition (EMT) is an essential process during embryogenesis and organ development in which cells transit from epithelial cells to those with the mesenchymal cells features [18,19]. EMT also plays a vital role in wound healing, tissue repair, organ fibrosis, cancer progression, and metastases. The EMT process has been shown to lead to loss of intercellular junctions, disrupt the epithelial polarity, and increase cell motility [20,21]. During the process, the increased expression of mesenchymal cell markers (e.g., vimentin, N-cadherin (N-cad), fibronectin) and the repression of epithelial cell markers (e.g., E-cadherin (E-cad), claudins, occludins) have been observed [22,23]. The EMT is also regulated by EMT-associated transcription factors such as Snail, Twist, and Zinc Finger E-box Binding (EZB) family [24]. The downregulation of E-cadherin is controlled by the transcriptional level by Snail protein [25]. Meanwhile, EMT confers metastatic and cancer stem cell properties, such as Sox-2, Oct-4, and CD133 to cancer cells and is always correlated with poor clinical outcomes for cancer patients with malignant transformation stage [26,27]. Thus, anti-EMT resistance drugs or multidrug therapies could be considered the prevention strategies for invasion and dissemination of tumor cells, to prevent the malignant cell migration, repress the cancer stemness, and gain the effectiveness of more classical chemotherapeutics.

Lung cancers are one of the most common carcinomas, which contain two main types, namely, small-cell lung cancer (SCLC) and non-small-cell lung cancer (NSCLC) [28]. Despite the advances of therapeutic treatment for tumor cells, the aggressiveness and metastatic potential of NSCLC remain a challenging topic to be overcome [29,30]. Emerging evidence suggests that the voltage-gated control is a check point of the cell cycle in cell proliferation [31]. Hence, we hypothesize that artificial depolarization or hyperpolarization might be used to discover novel mechanisms to control the cell cycle or proliferation of lung cancer cells. In this study, we evaluated the potential in modulating cancer cell proliferation, cell cycle, migration ability, and related gene expression by the photosensitive HEBR associated with ion pumping after light exposure.

## 2. Results

### 2.1. HEBR Protein Suppresses the Cell Proliferation of A549 and H1299 Cells

**Figure 1:**
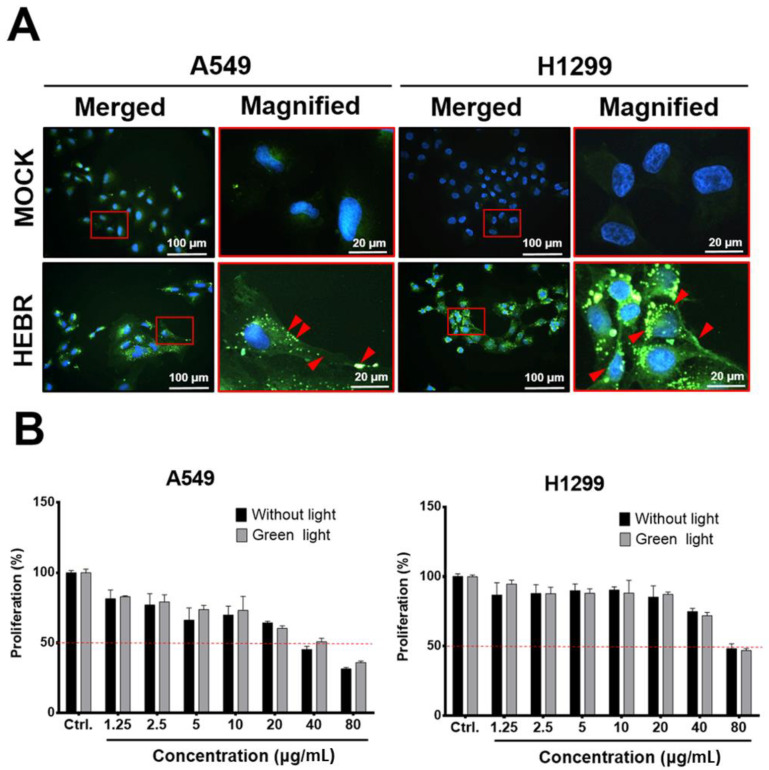
The effect of HEBR on the cell proliferation of A549 and H1299. (**A**) The distribution of HEBR protein expression on cell membranes and in the cytoplasm. Immunofluorescent staining HEBR was incubated with the HEBR proteins (20 μg/mL) on A549 or H1299 cells for 24 h. Immunofluorescence imaging of HEBR-his tag (green) counterstained with nucleic Hoechst (blue), and then merged. The magnified image of the selected region is shown in the red box. The HEBR proteins on cell membranes are demonstrated by red arrows. (**B**) The cell proliferation rate of A549 and H1299 after treatment with HEBR protein for 48 h evaluated by CCK-8 assay. The data are expressed as the relative proliferation of cells. The black bar represents the treatment of HEBR protein without green-light illumination, while the gray bar represents the treatment of HEBR with green-light illumination. The proliferation percentage at different HEBR concentrations was evaluated by GraphPad Prism 5 software.

Before light exposure to the HEBR-treated cells, we utilized immunofluorescent staining to confirm if the HEBR proteins could be delivered into A549 and H1299 cells, after incubation for 24 h with the culture medium containing 20 μg/mL of HEBR proteins. The data showed that HEBR proteins were obviously distributed on the cell membrane and in the cytoplasm of the stained cells, indicated by red arrows in the magnified images (Figure 1A). These data indicated that the effect of light stimulation on HEBR-treated cells should be evaluated after 24 h of incubation to ensure that the HEBR protein had entered cells. After light stimulation, the proliferation of the HEBR-treated cells was determined by CCK-8 assay (Figure 1B). The data showed that the proliferation of A549 and H1299 cells decreased in a dose-dependent manner after treatment of HEBR protein with or without green-light illumination. As shown in Figure 1B, the viability of A549 cells could be reduced below ~50% at the concentration of 40 μg/mL without the green-light exposure, while the proliferation of H1299 cells remained above 50% under the same condition. At the same time, a significantly decreased proliferation of H1299 cells was observed at the HEBR concentration of 80 μg/mL. For HEBR-treated cells with green-light illumination, the curve of A549 cells after treatment with HEBR protein was slightly different from those without light exposure. The results revealed no significantly differences between the green-light-illuminated or non-illuminated groups on either A549 or H1299 cells after the treatment of HEBR protein.

### 2.2. Cell Cycle Arrest in HEBR-Protein-Treated A549 Cells

**Figure 2:**
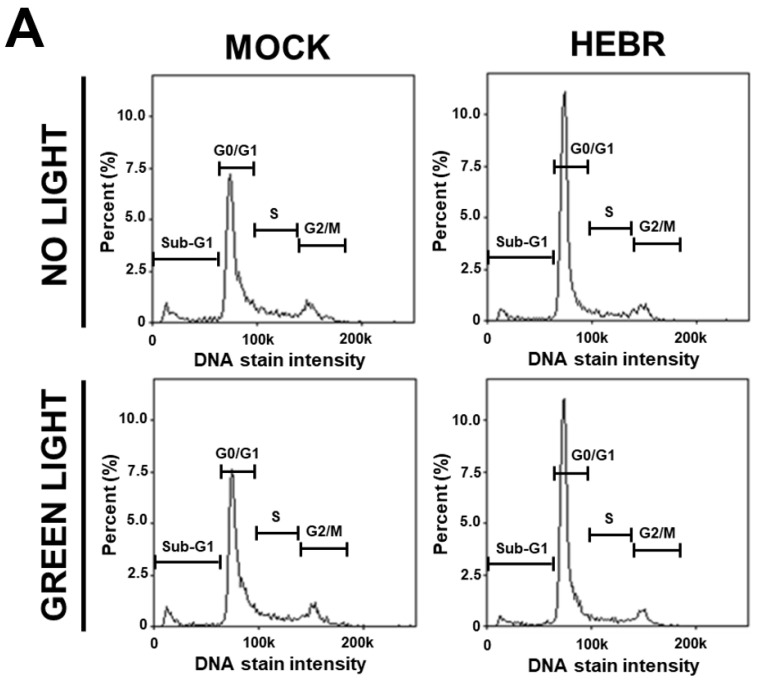

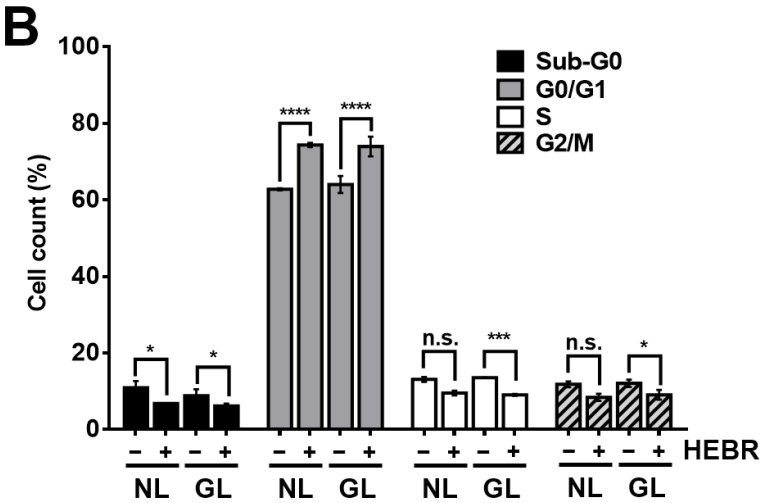
The induction of G_0_/G_1_ cell cycle arrest by HEBR. (**A**) The two-step cell cycle assay was used to detect the cell cycle distribution in A549. Cells were treated with HEBR protein (20 μg/mL) for 24 h with and without green-light illumination. Cells in different cell cycle stages were demarcated by the markers. (**B**) The markers of different cell cycle stages were transformed into histograms by GraphPad Prism 5 software. NL, no light illumination; GL, with green-light illumination. Asterisks indicate statistically significant differences, * *p* ≤ 0.05, *** *p* ≤ 0.001, **** *p* ≤ 0.0001.

To reveal the anti-proliferative mechanism of the HEBR protein, we assessed the effect of HEBR treatment on the cell cycle progression. Since the HEBR proteins had more growth-inhibitory effect on A549 cells than on H1299 cells. The A549 cells were selected for further investigation. A two-step cell cycle assay was carried out to quantify the distribution of DNA contents in different cell cycle stages under the concentration of 20 μg/mL HEBR in combination with (or without) green-light illumination on A549 (Figure 2A). Data showed the cell cycle G_0_/G_1_ phase arrest induced by the treatment of HEBR on A549 cells (Figure 2B). Meanwhile, no apparent differences between the green-light-illuminated or non-illuminated groups were observed in different cell cycle stages on either HEBR-treated or non-treated cells. The HEBR protein was found to promote high DNA contents at G_0_/G_1_ phase of the cell cycle with or without green-light exposure, indicating that A549 cells treated with HEBR protein induced cell cycle arrest at G_0_/G_1_ phase. These data suggested that HEBR protein could have growth-inhibitory effects on lung cancer cells without green-light exposure.

### 2.3. Scratch Assay on HEBR-Treated Cells

**Figure 3:**
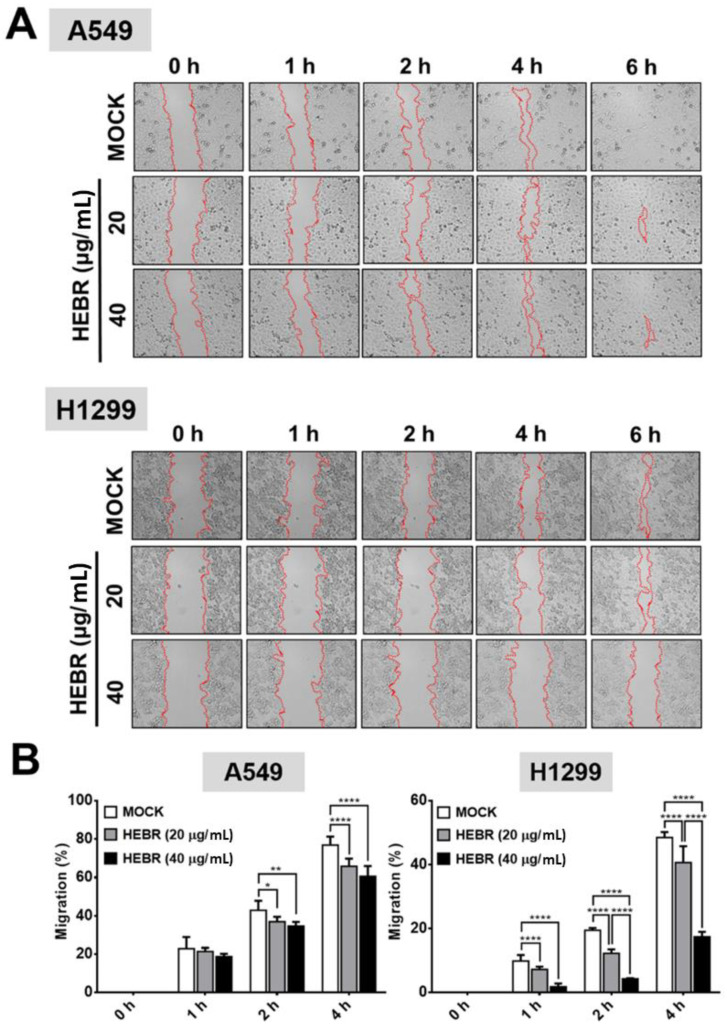
Inhibition of cell migration by HEBR for A549 and H1299 cells. To determine the effect of HEBR treatment on cell migration, a confluent monolayer of A549 and H1299 cells was scratched and then treated with HEBR (20 μg/mL or 40 μg/mL). After 6 h, the scratched area was observed, demonstrating subsided migration ability upon the treatment of HEBR. (**A**) The migration rate of A549 and H1299 treated with HEBR protein were recorded at 0 h, 1 h, 2 h, 4 h, and 6 h. (**B**) The area of migrated cells to the wound sides was evaluated. The percentage of cellular migration was calculated using the following formula: [(Area 0 h − Area determine h)/Area 0 h] × 100. The percentage of migration ability was calculated by GraphPad Prism 5 software. Data are presented as means ± SD. (n = 5); asterisks indicate statistically significant differences, * *p* ≤ 0.05, ** *p* ≤ 0.01, **** *p* ≤ 0.0001.

Migration assay is usually used in discovery of antitumor agents, as the agents that inhibit cell migration ability are potential anti-metastatic drugs. In this study, the scratch test was utilized to measure the migration ability of A549 and H1299 cells after treatment of HEBR protein (20 μg/mL or 40 μg/mL) without green-light illumination (Figure 3A) for a period of 6 h. The cellular migration of each HEBR-treated cells was quantified as a percentage and represented by bar diagrams at 0 h, 1 h, 2 h, and 4 h (Figure 3B). The HEBR protein did not reduce the migration ability of A549 cells within 6 h. The A549 cells treated with 40 μg/mL HEBR protein showed slightly decreased cell migration compared to A549 cells treated with 20 μg/mL HEBR protein within the 4 h of observation. HEBR protein at 20 μg/mL demonstrated a low inhibitory effect on the cell migration ability of H1299 and A549. Notably, the migration rate of H1299 cells was reduced to about 20% after HEBR treatment of 40 μg/mL for 4 h compare to un-treated cells (Figure 3B). The data showed that high concentrations of HEBR protein significantly inhibited the migration of H1299 cells rather than that of A549 cells, and the effect was clearly observed after 4 h of incubation time.

### 2.4. Transwell Assay of HEBR Protein on A549 and H1299 Cells

**Figure 4:**
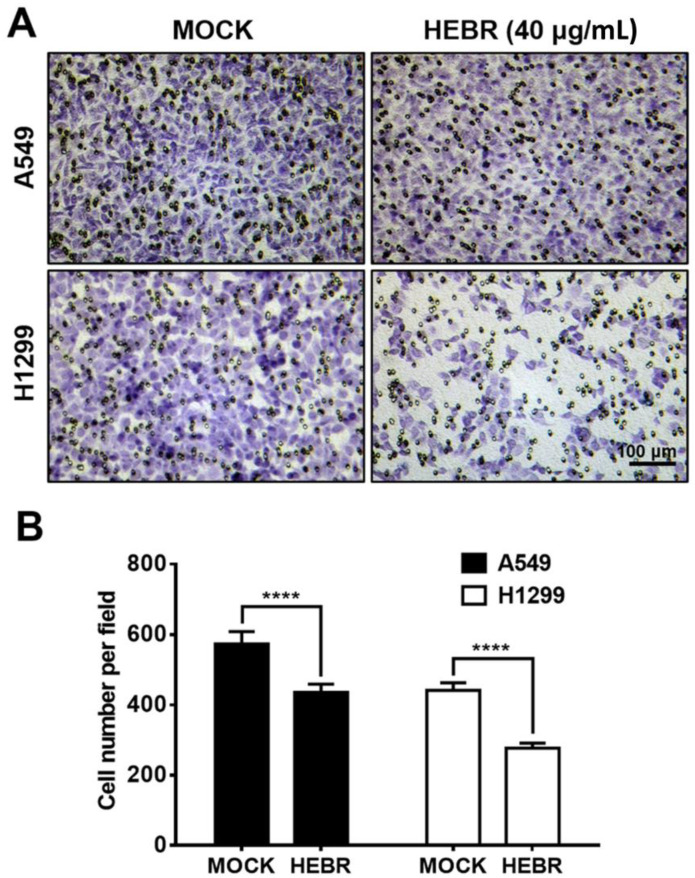
Inhibition of cell invasion by HEBR protein. (**A**) Transwell assay for the ability of A549 and H1299 invasion was conducted after treatment with HEBR (40 μg/mL) for 24 h. The number of A549 and H1299 cells invading through the transwell membrane significantly decreased upon the HEBR treatment. (**B**) The cell invasion ability was evaluated by counting cells per field using GraphPad Prism 5 software. Data are presented as means ± SD (n = 3~4). Asterisks indicate statistically significant differences, **** *p* ≤ 0.0001.

Since HEBR protein of 40 μg/mL showed a strong inhibitory effect on the migration of H1299 cells in the scratch assay, we further utilized a transwell migration assay to confirm the inhibitory effects on the cell migration of A549 and H1299 after treatment with HEBR (40 μg/mL) for 24 h (Figure 4). The results revealed that HEBR protein reduced the number of each type of lung cancer cells to pass through the transwell membrane compared to the non-treated cells (Figure 4A). It was evident that after HEBR protein treatment, H1299 cells had a lower number of migrated cells than A549 cells (Figure 4B). The transwell assay again proved that the dose of 40 μg/mL HEBR had a significantly inhibitory effect on the migration of H1299 cells.

### 2.5. Gene Expression of A549 and H1299 Cells after Treatment with HEBR Protein

**Figure 5:**
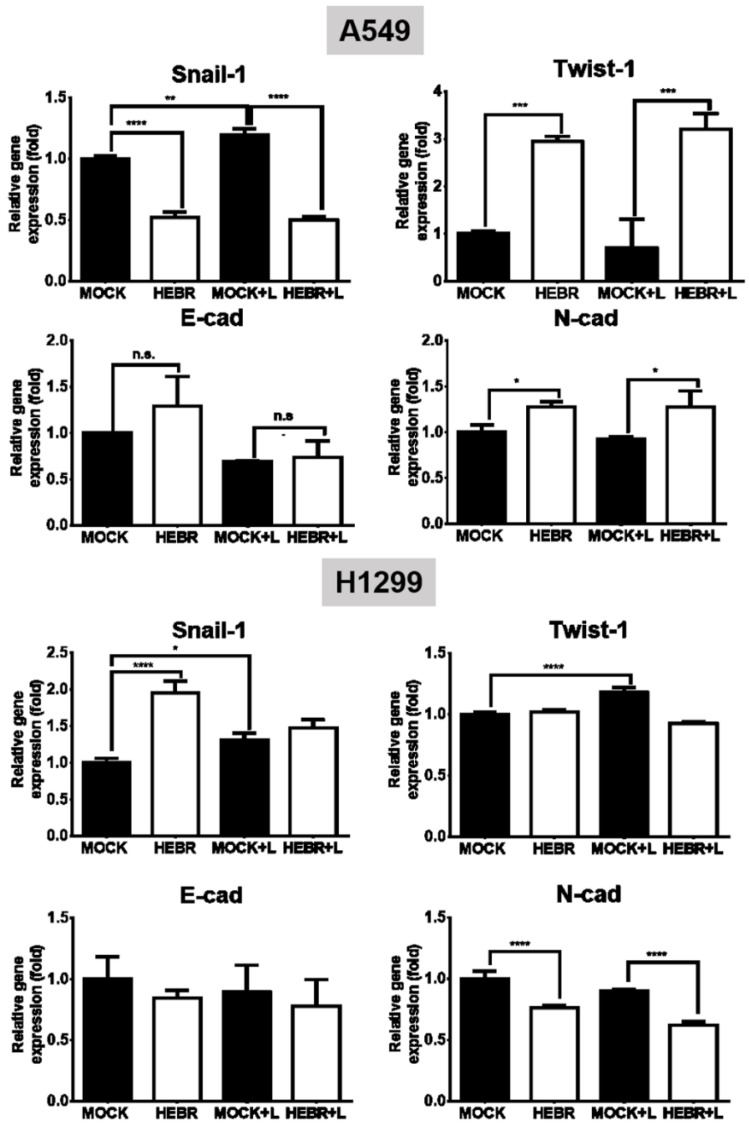
Gene expression of EMT HEBR treatment in A549 and H1299 cells. The cells were treated with HEBR proteins for 72 h with and without green-light illumination. The gene expression levels of markers for EMT, including Snail-1, Twist-1, E-cad, and N-cad, were analyzed for A549 or H1299 cells by qRT-PCR and calculated by GraphPad Prism 5 software. The expression of each marker was normalized to that of GAPDH in each group and then presented as the relative expression level to the mock group. L: treated with green-light illumination. Asterisks indicate statistically significant differences, * *p* ≤ 0.05, ** *p* ≤ 0.01, *** *p* ≤ 0.001, **** *p* ≤ 0.0001.

The mRNA expression levels of EMT genes (Snail-1, Twist-1, E-cad, and N-cad) and stemness genes (Sox-2, Oct-4, and CD133) for the illuminated or non-illuminated groups of HEBR-treated cells were quantified by qRT-PCR, and the results are shown in Figure 5. The gene expression level of Snail-1 for HEBR-protein-treated A549 cells was decreased either with or without green-light illumination (Figure 5). In contrast, the Twist-1 expression in A549 cells after treatment of HEBR protein was upregulated when compared with non-treated A549 cells. Meanwhile, HEBR protein did not significantly influence the expression levels of E-cad and N-cad for A549 cells under different conditions of light exposure.

**Figure 6:**
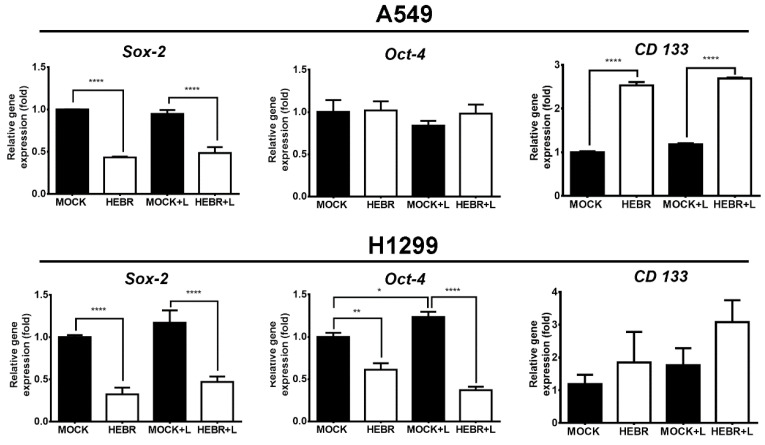
Gene expression of stemness after HEBR treatment in A549 and H1299 cells. The cells were treated with HEBR proteins for 72 h with and without green-light illumination. The gene expression levels of stemness markers included Sox-2, Oct-4, and CD133, for A549 and H1299, respectively. The expression of each marker was normalized to that of GAPDH in each group and then presented as the relative expression level to the mock group and analyzed by GraphPad Prism 5 software. L: treated with green-light illumination. Asterisks indicate statistically significant differences, * *p* ≤ 0.05, ** *p* ≤ 0.01, **** *p* ≤ 0.0001.

The expression of stemness-related genes in A549 and H1299 cells after treatment with HEBR protein with or without green-light exposure is displayed in Figure 6. The expression levels of Oct-4 did not display obvious difference between HEBR-treated and non-treated A549 cells. In addition, A549 cells in the non-treated groups expressed more Sox-2 gene than those in HEBR-treated groups. However, the expression level of CD133 in A549 cells was significantly increased in the HEBR-treated groups than that in the non-treated groups. The results showed that the HEBR protein significantly upregulated the expression of both Sox-2 and Oct-4 genes in H1299 cells, while increased the expression of CD133 (Figure 6). The expression of these stemness-related genes had no dramatic difference between green-light-illuminated groups and non-illuminated groups for both A549 and H1299 cells. These findings were consistent with the results of cell viability.

## 3. Discussion

The HEBR protein is a light-trigger proton pump that has optogenetic effects to regulate the function of ion flow artificially, which has been used to investigate new photo-modulable treatments via specific lighting stimulation on neural cells in our previous reports [16,32,33]. Modulation of light-triggered proteins has been reported to develop novel treatments based on light-driven control of neural activity in many optogenetic experiments [34]. For instance, the strategy of optogenetic activation increased the possible effects on serotonin nerve conduction to retrieve lost memory in Alzheimer’s disease through proton inflow [35]. Artificial manipulation of neural activity could induce the activation of glutamate receptors through action potentials for improving recognition memory in mouse models [36]. However, a few studies have developed photoactivatable cancer drug based on the function of ion channels under light control [37]. In many recent studies, optical control of intracellular signals was frequently used to design cancer drugs via regulation of signaling pathways [38,39,40]. Therefore, controllable ion channels might be an alternative optogenetic tool to develop antitumor drugs. In the present study, we used different concentrations of HEBR protein to evaluate the effect on lung cancer cells under different light-illuminated conditions. After treatment with proton ion pump proteins on A549 cells without light illumination, significant effects of growth inhibition on cancer cells by HEBR protein of the concentration of 40 μg/mL were observed. This is different from a previous study which demonstrated that HEBR-plasmid-transfected cells could pump out proton ion after green-light illumination [33]. No dramatic difference effects in the proliferation of A549 and H1299 cells was observed between the green-light group and the no-light group after the application of HEBR proteins. Thus, the effects of light-driven proton-pump activity may not play a major role in reducing the cell growth of the two types of cancer cells examined in this study. In addition, we noticed that the growth of human fibroblasts was not strongly affected by the treatment of HEBR protein under different conditions of light exposure (Figure S1), indicating that normal cells experienced less cytotoxic effects than the two types of cancer cells upon HEBR treatment.

Since the HEBR protein was more cytotoxic to A549 versus H1299 cells, we further investigated if the cell death of A549 was induced by HEBR-protein treatment under green-light stimulation. The cell cycle distribution showed that 20 μg/mL HEBR protein could induce cell cycle arrest at G_0_/G_1_ stage for A549 cells. However, there was no significant difference between light-illuminated and non-illuminated A549 cells after the treatment of 20 μg/mL HEBR protein for 24 h. This finding was consistent with the proliferation assay of A549 cells after the treatment of HEBR protein under different green-light-exposure conditions. These observations again suggested that the HEBR protein could cause growth-inhibitory effects on A549 cells without activation of the proton pump.

The metastasis of brain and bone is the leading cause of mortality in patients with lung cancer [41]. Wound healing and transwell assays are commonly used to quantify cellular migration ability. In the present study, we showed that HEBR effectively inhibited the migration of A549 and H1299 cells (Figure 3 and Figure 4). Besides, the epithelial-mesenchymal transition (EMT) program is a variable and dynamic multi-step process that occurs prior to tumor cell metastasis. Cancer cell metastasis relies on the migratory abilities of mesenchymal cells, which allow their entry into the surrounding blood vessels and lymph channels for distant metastasis [42]. In this study, HEBR significantly inhibited the migration ability of A549 and H1299 cells (Figure 3 and Figure 4), which suggested that HEBR may prevent cell metastasis by inhibiting the EMT program of lung cancer cells. When the EMT process starts, Snail is upregulated and E-cad is downregulated, both of which promote the shift of epithelial cells to a mesenchymal phenotype to increase tumor metastasis ability [43]. The HEBR treatment significantly upregulated the Twist-1 gene expression levels but downregulated the Snail-1 gene expression levels of A549 cells, which led to the decrease in cell migration ability. Meanwhile, the N-cad gene expression levels were downregulated, but the Snail-1 gene expression levels were upregulated in H1299 cells after the treatment of HEBR. These results suggested that HEBR might inhibit the EMT of A549 and H1299 cells by a different signaling pathway.

Indeed, the application of HEBR significantly reduced Sox-2 gene expression in A549 and H1299 cells. Many reports have suggested that SOX-2 plays an important role in cancer and cancer stem cells [44]. The downregulation of transcription factor SOX-2 in cancer stem cells (CSCs) was correlated with the suppression of lung cancer growth and metastasis behavior [45]. The SOX-2 knockdown in murine lung carcinoma cells inhibited tumor growth and metastases in C57BL/6 mice in vivo [45]. In gastric tumor cells, Sox-2 and Oct-4 play main roles in the proliferation, migration, invasion, and tumorigenicity [46]. The overexpression of Oct-4 and Sox-2 is linked to a greater migration ability [46]. In the current study, the gene expression of Sox-2 and Oct-4 decreased the HEBR-treated lung cancer cells, especially for H1299 cells (Figure 5). This result was consistent with the weaker migration ability observed in HEBR-treated H1299 cells compared with A549 cells (Figure 3 and Figure 4).

In summary, we showed that HEBR protein significantly inhibited the proliferation and migration of lung cancer cells in vitro regardless of whether the green light was employed. The phenomenon was positively correlated with the suppression of stemness of CSCs and EMT activity of the lung cancer cells.

## 4. Materials and Methods

### 4.1. Cell Culture

Human epithelial lung cancer cell lines (A549 and H1299) were cultured in the Roswell Park Memorial Institute medium (RPMI 1640, Gibco, Paisley, UK). Human dermal fibroblasts were isolated from the human foreskin (IRB#100-05-251) [32,47], which was maintained in the high-glucose Dulbecco’s modified Eagle medium (DMEM, Gibco, Grand Island, NY, USA). The two abovementioned culture media contained 10% fetal bovine serum (FBS, Gibco, Grand Island, NY, USA) and 1% Antibiotic-Antimycotic (Gibco, Grand Island, NY, USA). The cultured cells were incubated under the condition of 5% CO_2_ at a stable temperature of 37 °C.

### 4.2. Preparation of Purified HEBR Protein

HEBR-protein expression was established by the *Escherichia coli* (*E. coli*) system according to previous literature [15,16]. *E. coli* strain C43 (DE3) was used as the host cell to prepare the protein. The protein concentration was calculated by UV-VIS spectroscopy.

### 4.3. Immunostaining for HEBR Protein in A549 and H1299 Cells

Immunostaining assay was performed by following the procedures previously described [16]. Briefly, 7500 cells were seeded on each well of a 24-well plate overnight. The cells were then treated with HEBR protein (20 μg/mL) for 24 h. After incubation, the cells were washed with PBS and fixed with 4% paraformaldehyde (PFA) for 15 min. The cells were rinsed with PBS and blocked with 1% bovine serum albumin (BSA) for 1 h. Cells were then incubated with 6X His tag antibody (GTX628914, GeneTex, Hsinchu, Taiwan) at 4 °C overnight, followed by treatment of Alexa Fluor 488 anti-Rabbit (ab150080) secondary polyclonal antibody. Cell nuclei were counterstained with Hoechst 33258. After staining, the coverslip was mounted in Fluoro-Gel (Electron Microscopy Sciences, Hatfield, PA, USA), and the fluorescence was observed by a fluorescence microscope (Nikon EClipse 80i, Tokyo, Japan).

### 4.4. Cell Viability Analysis

The cell viability of A549 or H1299 treated with HEBR protein was further analyzed by the Cell Counting Kit-8 (Dojindo, Kumamoto, Japan) assay according to the manufacturer’s instructions [16]. A549 or H1299 cells with a density of 1500 cells/well were seeded into 96-well culture plates for 24 h and incubated with several concentrations of HEBR protein in a culture RPMI medium. After incubation for 24 h, the cells were stimulated with (or without) the green (525 nm)-light-emitting diode (LED) for three times per day. Each illumination lasted for 10 s, and the exposure distance between the light source and cultured cell lines was ~2 cm. After incubation for 48 h, the medium was switched to the culture medium with 100 μL of the CCK-8 reagent to obtain orange-colored solution. After incubation for 60 min, absorbance at the wavelength of 450 nm was detected. The percentage of cell viability was measured as the percentage of viable cells relative to untreated cells.

### 4.5. Cell Cycle

The cell cycle of A549 was performed as previously described [16]. Briefly, A549 cells were cultured under the serum-deprived RPMI medium for 18 h to synchronize the cell cycle before the HEBR protein treatment. The cells were incubated to the complete culture RPMI medium containing 40 μg/mL HEBR proteins for 24 h. The profile of cell cycle of A549 was determined by a two-step cell cycle assay (Chemometec, Allerød, Denmark) using the NucleoCounter^®^ NC-3000^TM^ instrument according to the manufacturer’s instructions.

### 4.6. Migration Assay

The migration ability of A549 or H1299 after the treatment treated of HEBR protein was evaluated by the scratch-migration assay and transwell migration assay as previously described [48,49]. For the scratch-migration assay, the cells (2.5 × 10^6^ cells/well) were seeded in 24-well plates for 24 h and a gap region was made using a sterile 200 µL pipette tip across the cell monolayer (>90% confluent). HEBR proteins of two different concentrations were incubated with the cells and the gap region was tracked by a time-lapse recording system (Real Time Cell Culture Monitoring (CCM) System, Astec, Fukuoka, Japan ) in RPMI medium. A series of the scratching images was captured from 0 h to 6 h. Quantification of migration rate was carried out using the MRI wound healing tool of NIH Image J software. For transwell migration assay, 4 × 10^5^ cells were seeded on each 24 mm-diameter transwell membrane (0.8 μm pore size, Corning, NY, USA) with 1.5 mL serum-free RPMI medium in the upper chamber. In the lower chamber, 2 mL basal culture RPMI medium with 10% FBS was used as a chemoattractant for cells to promote cell migration. Cells were treated with HEBR (20 μg/mL) and incubated for 24 h. The cells that did not migrate were removed by a cotton swab and stained with crystal violet (0.2%). The images of migrated cells were captured under an inverted microscope and the quantification of the cell number was conducted by the cell-counter tool of Image J software.

### 4.7. RNA Extraction and Quantitative Real-Time Reverse Transcription Polymerase Chain Reaction (qRT-PCR) Analysis

The experiments were carried out as previously described [16]. The total RNA of the A549 or H1299 cells was isolated using the Trizol reagent (Invitrogen, Carlsbad, CA, USA) after the treatment of the HEBR (40 μg/mL) with or without light illumination for 48 h. The RNA strands were extracted by RevertAid™ First Strand cDNA Synthesis Kit (Thermo Scientific, Vilnius, Lithuania) to synthesize complementary DNA (cDNA). The gene expression level was labeled by the KAPA SYBR green fast qPCR Master Mix Kit (KAPA Biosystems, Cape Town, South Africa) and performed on a StepOnePlus™ Real-Time PCR System (Applied Biosystems, Singapore). The EMT and stemness gene-expression markers such as Snail-1, Twist-1, E-cad, N-cad, Sox-2, Oct-4, and CD133 were evaluated after the treatment of HEBR protein. The expression levels were normalized by endogenous housekeeping gene glyceraldehyde 3-phosphate dehydrogenase (GAPDH).

### 4.8. Statistical Analyses

The experimental data were displayed as the mean ± standard deviation (SD). Each quantitative experiment was independently performed three times for each group. A one-way ANOVA test was conducted to examine the statistical difference for two or more experimental groups, using GraphPad Prism 5 software.

## 5. Conclusions

This study demonstrated that the optogenetic HEBR protein significantly influenced lung cancer cell proliferation and cell cycle progression, reduced migration activity, and suppressed some stemness-related genes. The administration of HEBR has a substantially different effect on cancer cells compared to normal cells, which could serve as a novel therapeutic strategy for the treatment of lung cancer metastasis. In the current study, we obtained the optimal concentration range of HEBR to repress the migration activity of cancer cells. In the future, we will use the mice model and combine multidrug treatment to further investigate the effect of HEBR on the progression and migration behavior of tumor. Meanwhile, Sox-2 and Oct-4 are well known to be involved in maintaining the stemness, cancer progression, and the resistance towards cancer therapies of cancer cells. Therefore, HEBR protein may be combined with cancer drugs to suppress the Sox-2 and Oct-4 gene expression of lung cancer stem cells. Taken together, we propose that the HEBR protein may be useful in cancer therapeutics, and the control mechanism of cancer cell migration ability may be independent of light exposure.

## Data Availability

Not applicable.

## Supplementary Materials

Figure S1: Cell viability of human fibroblasts after treatment with HEBR protein for 72 h.

## References

[1] Rodríguez-Valera F. (1993). Introduction to Saline Environments. The Biology of Halophilic Bacteria.

[2] Ventosa A., Nieto J.J., Oren A. (1998). Biology of moderately halophilic aerobic bacteria. Microbiol. Mol. Biol. Rev..

[3] Sharma A.K., Walsh D.A., Bapteste E., Rodriguez-Valera F., Doolittle W.F., Papke R.T. (2007). Evolution of rhodopsin ion pumps in haloarchaea. BMC Evol. Biol..

[4] Oesterhelt D., Stoeckenius W. (1971). Rhodopsin-like protein from the purple membrane of *Halobacterium halobium*. Nat. New Biol..

[5] Ernst O.P., Lodowski D.T., Elstner M., Hegemann P., Brown L.S., Kandori H. (2014). Microbial and animal rhodopsins: Structures, functions, and molecular mechanisms. Chem. Rev..

[6] Mukhina T., Gerelli Y., Hemmerle A., Koutsioubas A., Kovalev K., Teulon J.-M., Pellequer J.-L., Daillant J., Charitat T., Fragneto G. (2021). Insertion and activation of functional Bacteriorhodopsin in a floating bilayer. J. Colloid Interface Sci..

[7] Schobert B., Lanyi J.K. (1982). Halorhodopsin is a light-driven chloride pump. J. Biol. Chem..

[8] Feroz H., Ferlez B., Oh H., Mohammadiarani H., Ren T., Baker C.S., Gajewski J.P., Lugar D.J., Gaudana S.B., Butler P. (2021). Liposome-based measurement of light-driven chloride transport kinetics of halorhodopsin. Biochim. Biophys. Acta BBA Biomembr..

[9] Kovalev K., Polovinkin V., Gushchin I., Alekseev A., Shevchenko V., Borshchevskiy V., Astashkin R., Balandin T., Bratanov D., Vaganova S. (2019). Structure and mechanisms of sodium-pumping KR2 rhodopsin. Sci. Adv..

[10] Nagel G., Szellas T., Huhn W., Kateriya S., Adeishvili N., Berthold P., Ollig D., Hegemann P., Bamberg E. (2003). Channelrhodopsin-2, a directly light-gated cation-selective membrane channel. Proc. Natl. Acad. Sci. USA.

[11] Kato H.E., Yawo H., Kandori H., Koizumi A., Kageyama R. (2021). Structure–function relationship of channelrhodopsins. Optogenetics.

[12] Kühlbrandt W. (2000). Bacteriorhodopsin—The movie. Nature.

[13] Miller R.D., Paré-Labrosse O., Sarracini A., Besaw J.E. (2020). Three-dimensional view of ultrafast dynamics in photoexcited bacteriorhodopsin in the multiphoton regime and biological relevance. Nat. Commun..

[14] Tu C.-H., Yi H.-P., Hsieh S.-Y., Lin H.-S., Yang C.-S. (2018). Overexpression of different types of microbial rhodopsins with a highly expressible bacteriorhodopsin from *Haloarcula marismortui* as a single protein in *E. coli*. Sci. Rep..

[15] Hsu M.-F., Yu T.-F., Chou C.-C., Fu H.-Y., Yang C.-S., Wang A.H.J. (2013). Using *Haloarcula marismortui* Bacteriorhodopsin as a fusion tag for enhancing and visible expression of integral membrane proteins in *Escherichia coli*. PLoS ONE.

[16] Han H.-W., Ko L.-N., Yang C.-S., Hsu S.-h. (2019). Potential of engineered bacteriorhodopsins as photoactivated biomaterials in modulating neural stem cell behavior. ACS Biomater. Sci. Eng..

[17] Mattingly M., Weineck K., Costa J., Cooper R.L. (2018). Hyperpolarization by activation of halorhodopsin results in enhanced synaptic transmission: Neuromuscular junction and CNS circuit. PLoS ONE.

[18] Mittal V. (2016). Epithelial mesenchymal transition in aggressive lung cancers. Lung Cancer and Personalized Medicine: Novel Therapies and Clinical Management.

[19] Lim J., Thiery J.P. (2012). Epithelial-mesenchymal transitions: Insights from development. Development.

[20] Ribatti D., Tamma R., Annese T. (2020). Epithelial-mesenchymal transition in cancer: A historical overview. Transl. Oncol..

[21] Brabletz T., Kalluri R., Nieto M.A., Weinberg R.A. (2018). EMT in cancer. Nat. Rev. Cancer.

[22] Yan B., Zhang W., Jiang L.-Y., Qin W.-X., Wang X. (2014). Reduced E-Cadherin expression is a prognostic biomarker of non-small cell lung cancer: A meta-analysis based on 2395 subjects. Int. J. Clin. Exp. Med..

[23] Loh C.-Y., Chai J.Y., Tang T.F., Wong W.F., Sethi G., Shanmugam M.K., Chong P.P., Looi C.Y. (2019). The E-cadherin and N-cadherin switch in epithelial-to-mesenchymal transition: Signaling, therapeutic implications, and challenges. Cells.

[24] Georgakopoulos-Soares I., Chartoumpekis D.V., Kyriazopoulou V., Zaravinos A. (2020). EMT factors and metabolic pathways in cancer. Front. Oncol..

[25] Cano A., Pérez-Moreno M.A., Rodrigo I., Locascio A., Blanco M.J., del Barrio M.G., Portillo F., Nieto M.A. (2000). The transcription factor snail controls epithelial–mesenchymal transitions by repressing E-cadherin expression. Nat. Cell Biol..

[26] Lambert A.W., Weinberg R.A. (2021). Linking EMT programmes to normal and neoplastic epithelial stem cells. Nat. Rev. Cancer.

[27] Saigusa S., Tanaka K., Toiyama Y., Yokoe T., Okugawa Y., Ioue Y., Miki C., Kusunoki M. (2009). Correlation of CD133, OCT4, and SOX2 in rectal cancer and their association with distant recurrence after chemoradiotherapy. Ann. Surg. Oncol..

[28] Hirsch F.R., Scagliotti G.V., Mulshine J.L., Kwon R., Curran W.J., Wu Y.-L., Paz-Ares L. (2017). Lung cancer: Current therapies and new targeted treatments. Lancet.

[29] Jones G.S., Baldwin D.R. (2018). Recent advances in the management of lung cancer. Clin. Med..

[30] Jiang L., Huang J., Jiang S., Rong W., Shen Y., Li C., Tian Y., Ning J., Chen X., Yang Y. (2021). The surgical perspective in neoadjuvant immunotherapy for resectable non-small cell lung cancer. Cancer Immunol. Immunother..

[31] Serrano-Novillo C., Capera J., Colomer-Molera M., Condom E., Ferreres J.C., Felipe A. (2019). Implication of voltage-gated potassium channels in neoplastic cell proliferation. Cancers.

[32] Luo P.-W., Han H.-W., Yang C.-S., Shrestha L.K., Ariga K., Hsu S.-h. (2019). Optogenetic modulation and reprogramming of bacteriorhodopsin-transfected human fibroblasts on self-assembled fullerene C60 nanosheets. Adv. Biosyst..

[33] Hsieh F.-Y., Han H.-W., Chen X.-R., Yang C.-S., Wei Y., Hsu S.-h. (2018). Non-viral delivery of an optogenetic tool into cells with self-healing hydrogel. Biomaterials.

[34] Kozin E.D., Brown M.C., Lee D.J., Stankovic K.M. (2020). Light-based neuronal activation: The future of cranial nerve stimulation. Otolaryngol. Clin. N. Am..

[35] Bostanciklioglu M. (2020). Optogenetic stimulation of serotonin nuclei retrieve the lost memory in Alzheimer’s disease. J. Cell. Physiol..

[36] Wang K.W., Ye X.L., Huang T., Yang X.F., Zou L.Y. (2019). Optogenetics-induced activation of glutamate receptors improves memory function in mice with Alzheimer’s disease. Neural Regen. Res..

[37] Kielbus M., Czapinski J., Odrzywolski A., Stasiak G., Szymanska K., Kalafut J., Kos M., Giannopoulos K., Stepulak A., Rivero-Muller A. (2018). Optogenetics in cancer drug discovery. Expert Opin. Drug Discov..

[38] Hu W., Li Q., Li B., Ma K., Zhang C., Fu X. (2020). Optogenetics sheds new light on tissue engineering and regenerative medicine. Biomaterials.

[39] Zhang M., Lin X., Zhang J., Su L., Ma M., Ea V.L., Liu X., Wang L., Chang J., Li X. (2020). Blue light-triggered optogenetic system for treating uveal melanoma. Oncogene.

[40] McCormick J.W., Pincus D., Resnekov O., Reynolds K.A. (2019). Strategies for engineering and rewiring kinase regulation. Trends Biochem. Sci..

[41] D’Antonio C., Passaro A., Gori B., Del Signore E., Migliorino M.R., Ricciardi S., Fulvi A., De Marinis F. (2014). Bone and brain metastasis in lung cancer: Recent advances in therapeutic strategies. Ther. Adv. Med. Oncol..

[42] Talmadge J.E., Fidler I.J. (2010). AACR centennial series: The biology of cancer metastasis: Historical perspective. Cancer Res..

[43] Batlle E., Sancho E., Francí C., Domínguez D., Monfar M., Baulida J., De Herreros A.G. (2000). The transcription factor snail is a repressor of *E-cadherin* gene expression in epithelial tumour cells. Nat. Cell Biol..

[44] Hüser L., Novak D., Umansky V., Altevogt P., Utikal J. (2018). Targeting SOX2 in anticancer therapy. Expert Opin. Ther. Targets.

[45] Xiang R., Liao D., Cheng T., Zhou H., Shi Q., Chuang T., Markowitz D., Reisfeld R., Luo Y. (2011). Downregulation of transcription factor SOX2 in cancer stem cells suppresses growth and metastasis of lung cancer. Br. J. Cancer.

[46] Chen B., Zhu Z., Li L., Ye W., Zeng J., Gao J., Wang S., Zhang L., Huang Z. (2019). Effect of overexpression of *Oct4* and *Sox2* genes on the biological and oncological characteristics of gastric cancer cells. OncoTargets Ther..

[47] Lin Y.-H., Fu K.-Y., Hong P.-D., Ma H., Liou N.-H., Ma K.-H., Liu J.-C., Huang K.-L., Dai L.-G., Chang S.-C. (2013). The Effects of Microenvironment on Wound Healing by Keratinocytes Derived From Mesenchymal Stem Cells. Ann. Plast. Surg..

[48] Cormier N., Yeo A., Fiorentino E., Paxson J. (2015). Optimization of the wound scratch assay to detect changes in murine mesenchymal stromal cell migration after damage by soluble cigarette smoke extract. J. Vis. Exp..

[49] Chan C.-H., Lee S.-W., Li C.-F., Wang J., Yang W.-L., Wu C.-Y., Wu J., Nakayama K.I., Kang H.-Y., Huang H.-Y. (2010). Deciphering the transcriptional complex critical for *RhoA* gene expression and cancer metastasis. Nat. Cell Biol..

