# Supplementary material for: Engineered Bacteriorhodopsin May Induce Lung Cancer Cell Cycle Arrest and Suppress Their Proliferation and Migration
Source: Molecules. 2021 Dec 3;26(23):7344. doi: 10.3390/molecules26237344 (PMC8659022; doi:10.3390/molecules26237344)
Supplement: Supplementary file 1 [file molecules-26-07344-s001.zip › molecules-1488886-supplementary.pdf]

Supplementary figure

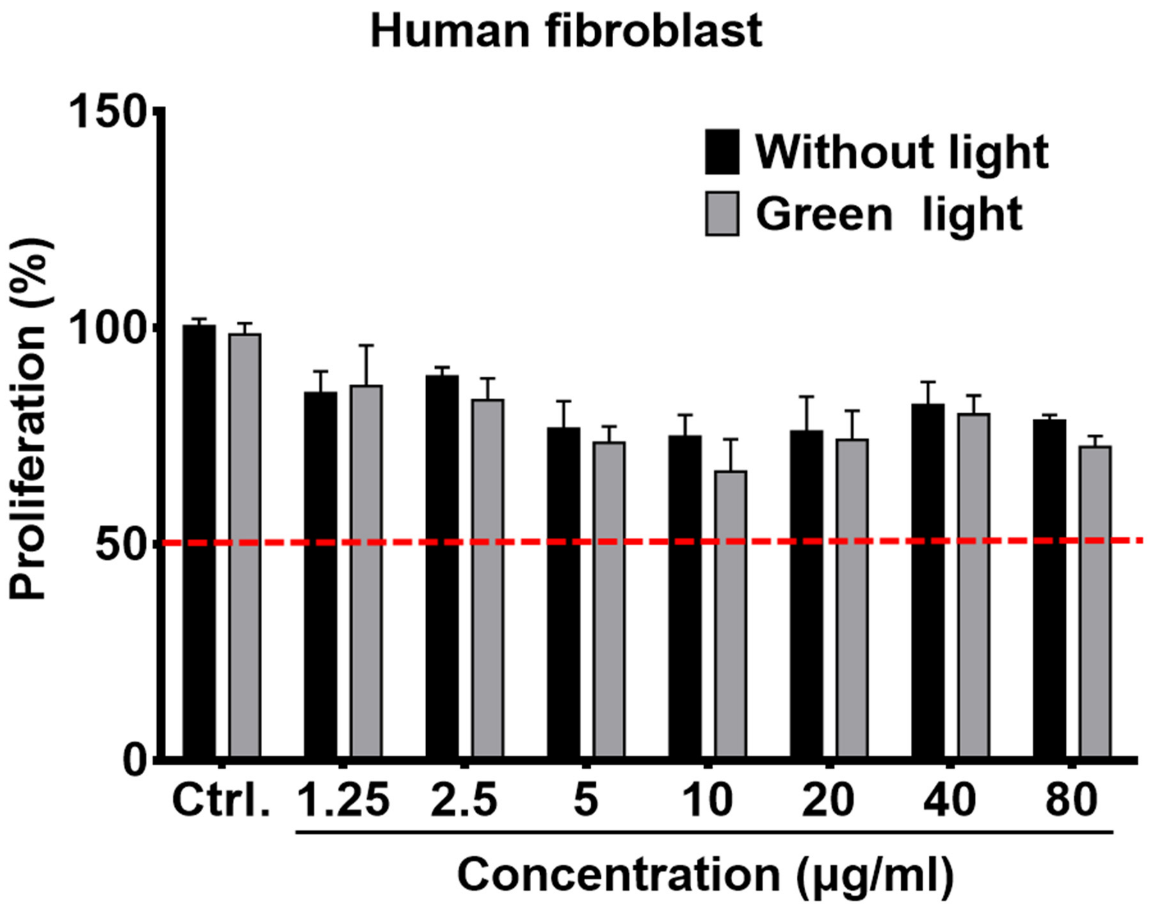

Figure S1. Cell proliferation of human fibroblasts after treatment with HEBR protein for 48 h. The data were expressed as the relative proliferation of cells by the CCK-8 assay. The human fibroblast viability was only slightly affected by the treatment of HEBR protein (80 μg/ml) either with or without green light illumination.
